# WW Domain-Containing E3 Ubiquitin Protein Ligase 1: A Self-Disciplined Oncoprotein

**DOI:** 10.3389/fcell.2021.757493

**Published:** 2021-10-12

**Authors:** Linghan Kuang, Yunhui Jiang, Chenghua Li, Yongmei Jiang

**Affiliations:** ^1^Department of Laboratory Medicine, West China Second University Hospital, Sichuan University, Chengdu, China; ^2^Key Laboratory of Birth Defects and Related Diseases of Women and Children (Sichuan University), Ministry of Education, Chengdu, China; ^3^Pathology Department, The Second People’s Hospital of Jingmen, Jingmen, China; ^4^Center of Growth, Metabolism and Aging, Key Laboratory of Biological Resources and Ecological Environment of Ministry of Education, College of Life Sciences, Sichuan University, Chengdu, China

**Keywords:** WW domain-containing E3 ubiquitin protein ligase 1 (WWP1), tumorigenesis and progression, protein degradation, ubiquitination, C2-WW-HECT E3 ligase family, transforming growth factor-beta (TGFβ), epidermal growth factor receptor (EGFR), Wnt/b-catenin

## Abstract

WW domain-containing E3 ubiquitin protein ligase 1 (WWP1) is a member of C2-WW-HECT E3 ligase family. Although it may execute carcinostatic actions in some scenarios, WWP1 functions as an oncoprotein under most circumstances. Here, we comprehensively review reports on regulation of WWP1 and its roles in tumorigenesis. We summarize the WWP1-mediated ubiquitinations of diverse proteins and the signaling pathways they involved, as well as the mechanisms how they affect cancer formation and progression. According to our analysis of database, in combination with previous reports, we come to a conclusion that WWP1 expression is augmented in various cancers. Gene amplification, as well as expression regulation mediated by molecules such as non-coding RNAs, may account for the increased mRNA level of WWP1. Regulation of enzymatic activity is another important facet to upregulate WWP1-mediated ubiquitinations. Based on the published data, we conclude that WWP1 employs interactions between multiple domains to autoinhibit its polyubiquitination activity in a steady state. Association of some substrates can partially release certain autoinhibition-related domains and make WWP1 have a moderate activity of polyubiquitination. Some cancer-related mutations can fully disrupt the inhibitory interactions and make WWP1 hyperactive. High expression level or hyperactivation of WWP1 may abnormally enhance polyubiquitinations of some oncoproteins or tumor suppressors, such as ΔNp63α, PTEN and p27, and ultimately promote cell proliferation, survival, migration and invasion in tumorigenesis. Given the dysregulation and oncogenic functions of WWP1 in some cancer types, it is promising to explore some therapeutic inhibitors to tune down its activity.

## Introduction

WW domain-containing E3 ubiquitin protein ligase 1 (WWP1) is also known as AIP5 (Atropin-1-interacting protein 5) or TIUL1 (TG-interacting ubiquitin ligase 1) (Wood et al., 1998; Seo et al., 2004; Zhi and Chen, 2012). It belongs to the C2-WW-HECT E3 ligase family, which also contains 8 extra members, i.e., WWP2 (also known as AIP2) (Zhang et al., 2019), NEDD4 (neural precursor cell expressed developmentally downregulated protein 4, also known as NEDD4-1) (Huang et al., 2019), NEDD4L (NEDD4-like ubiquitin protein ligase, also known as NEDD4-2) (Goel et al., 2015), NEDL1 (NEDD4-like ubiquitin protein ligase-1) (Miyazaki et al., 2004), NEDL2 (Wei et al., 2015), Itch (named in reference to skin-scratching behavior in mice lacking this protein, also known as Itchy or AIP4) (Perry et al., 1998; Yin et al., 2020), SMURF1 (Smad ubiquitination regulatory factor 1), and SMURF2 (Zhi and Chen, 2012; Fu et al., 2020). Some of these members may be functionally redundant with WWP1, given that WWP1 knockout mice are viable and fertile with no obvious abnormalities (Chen and Matesic, 2007; Shu et al., 2013). *WWP1* is highly expressed in multiple tissues (Wood et al., 1998; Komuro et al., 2004), where it can ubiquitinate plenty of proteins and regulate diverse cellular processes including protein trafficking, degradation, and cell signal transduction. Thus, this E3 ligase should be finely regulated, because dysregulation of it is involved in a variety of diseases, such as malignancies, cardiovascular diseases, and immune disorders (Zhi and Chen, 2012). Vast evidence reveals that WWP1 is overexpressed in multiple cancer types, especially some breast and prostate cancers, while downregulated in several classes of carcinomas. In these tumor tissues, WWP1 either promotes or inhibits tumorigenesis via modulating the protein levels or functions of its substrates (Zhi and Chen, 2012).

## The *WW Domain-Containing E3 Ubiquitin Protein Ligase 1* Gene and Its Expression

**FIGURE 1:**
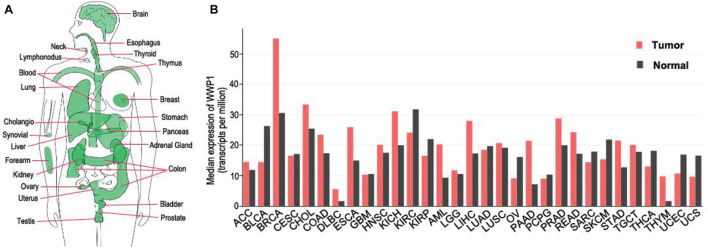
Expression of WWP1 in human body and tumor tissues. **(A)** WWP1 expression of normal samples in bodymap. All organs expressing WWP1 mRNA are painted green. WWP1 expression is analyzed in website http://gepia.cancer-pku.cn/. **(B)** The gene expression profile across all tumor samples and paired normal tissues according to the analysis in website http://gepia.cancer-pku.cn/. TPM, transcripts per million; ACC, Adrenocortical carcinoma; BLCA, Bladder Urothelial Carcinoma; BRCA, Breast invasive carcinoma; CESC, Cervical squamous cell carcinoma and endocervical adenocarcinoma; CHOL, Cholangio carcinoma; COAD, Colon adenocarcinoma; DLBC, Lymphoid Neoplasm Diffuse Large B-cell Lymphoma; ESCA, Esophageal carcinoma; GBM, Glioblastoma multiforme; HNSC, Head and Neck squamous cell carcinoma; KICH, Kidney Chromophobe; KIRC, Kidney renal clear cell carcinoma; KIRP, Kidney renal papillary cell carcinoma; AML, Acute Myeloid Leukemia; LGG, Brain Lower Grade Glioma; LIHC, Liver hepatocellular carcinoma; LUAD, Lung adenocarcinoma; LUSC, Lung squamous cell carcinoma; MESO, Mesothelioma; OV, Ovarian serous cystadenocarcinoma; PAAD, Pancreatic adenocarcinoma; PCPG, Pheochromocytoma and Paraganglioma; PRAD, Prostate adenocarcinoma; READ, Rectum adenocarcinoma; SARC, Sarcoma; SKCM, Skin Cutaneous Melanoma; STAD, Stomach adenocarcinoma; TGCT, Testicular Germ Cell Tumors; THCA, Thyroid carcinoma; THYM, Thymoma; UCEC, Uterine Corpus Endometrial Carcinoma; UCS, Uterine Carcinosarcoma.

The *WWP1* gene is highly conserved from *C. elegance* to mammals (Huang et al., 2000). It locates on chromosome 8q21 and spans up to 142 kilobase pairs, containing 26 exons (Malbert-Colas et al., 2003). Numerous somatic mutations occur in the *WWP1* gene in different human cancers (Wang et al., 2019). According to our analysis using GEPIA database^1^ (Tang et al., 2017), WWP1 mRNA is expressed in diverse tissues, such as brain, esophagus, breast, lung, liver, stomach, colon, prostate, and testis (Figure 1A). This is consistent with previous reports (Wood et al., 1998; Komuro et al., 2004). It has been reported that about 1 out of 2 or 3 prostate and breast cancers bear multiple copies of the *WWP1* gene due to gene amplification, resulting in an elevated expression level of this gene (Chen et al., 2007a,b; Nguyen Huu et al., 2008). Here, we comprehensively analyzed the expression profile of *WWP1* in different cancer types using GEPIA database (Tang et al., 2017). As shown in Figure 1B, besides breast invasive carcinoma (BRCA) and prostate adenocarcinoma (PRAD), WWP1 mRNA level is significantly upregulated in cholangio carcinoma (CHOL), colon adenocarcinoma (COAD), esophageal carcinoma (ESCA), kidney chromophobe (KICH), acute myeloid leukemia (AML), liver hepatocellular carcinoma (LIHC), pancreatic adenocarcinoma (PAAD), rectum adenocarcinoma (READ), stomach adenocarcinoma (STAD), and thymoma (THYM). Gene amplification may be the main cause of the high expression level of WWP1 in these malignancies, where this E3 ligase probably executes oncogenic functions. On the other hand, WWP1 is downregulated in bladder urothelial carcinoma (BLCA), kidney renal clear cell carcinoma (KIRC), kidney renal papillary cell carcinoma (KIRP), ovarian serous cystadenocarcinoma (OV), uterine corpus endometrial carcinoma (UCEC), and uterine carcinosarcoma (UCS). The undermentioned regulation mechanisms may account for the reduced expression of WWP1, which is likely to function as a tumor-suppressive E3 ligase in these cancers.

Expression of WWP1 is regulated by multiple mechanisms. It was reported that transforming growth factor β (TGFβ) stimulates transcription of *WWP1* gene via an unknown mechanism (Chen and Matesic, 2007). This suggests that TGFβ and WWP1 form a feedback loop, since WWP1 can repress TGFβ signaling via downregulating several components of this cascade (Komuro et al., 2004; Seo et al., 2004). Tumor necrosis factor α (TNFα) can also promote expression of WWP1 at mRNA level (Zhao et al., 2011). Data from Ceshi Chen group suggest that p53 positively regulates expression of WWP1 (Li Y. et al., 2008). In combination with the evidence that WWP1 impairs transactivity of p53 (Laine and Ronai, 2007), this indicates another feedback loop. According to data from Ceshi Chen group, DNA damage drugs induce expression of WWP1 via enhanced p53 level (Li Y. et al., 2008). Data from our group suggest that DNA damage may stimulate transcription of WWP1 through a p53-dependent manner or a miR-452-involved mode (Chen et al., 2017). Pier Paolo Pandolfi group recently reported that MYC directly binds to the promoter of WWP1 gene and activates its transcription (Lee et al., 2019). Besides miR-452 (Goto et al., 2016; Chen et al., 2017), several non-coding RNAs have been found to regulate expression of WWP1: microRNAs, including miR-15b (Li et al., 2020), miR-21 (Tao et al., 2018), miR-30a-5p (Zhao et al., 2019), miR-129-5p and -3p (Ma et al., 2018), miR-142 (Tu et al., 2017; Wang et al., 2021), and miR-584-5p (Li et al., 2017), inhibit WWP1 expression likely via destabilizing the WWP1 mRNA; long non-coding RNA SNHG12 and circular RNA circWAC sponge miR-129-5p and miR-142, respectively, to derepress the expression of WWP1 (Li et al., 2019; Wang et al., 2021). All the known factors regulating WWP1 expression at the mRNA level are summarized in Table 1. These data indicate that multiple signals, transcription factors, and non-coding RNAs may affect tumorigenesis via modulating expression of WWP1.

**TABLE 1:** Factors regulating WWP1 mRNA levels.

Factor	Molecule type	Effect on *WWP1* mRNA level	Mechanism	References
TGFβ	Extracellular signaling molecule	↑	unknown	Chen and Matesic, 2007
TNFα	Extracellular signaling molecule	↑	unknown	Zhao et al., 2011
p53	Transcription factor	↑	transactivation	Li Y. et al., 2008
MYC	Transcription factor	↑	transactivation	Lee et al., 2019
miR-452	microRNA	↓	Destabilizing *WWP1* mRNA	Goto et al., 2016
miR-15b	microRNA	↓	Destabilizing *WWP1* mRNA	Li et al., 2020
miR-21	microRNA	↓	Destabilizing *WWP1* mRNA	Tao et al., 2018
miR-30a-5p	microRNA	↓	Destabilizing *WWP1* mRNA	Zhao et al., 2019
miR-129-5p/3p	microRNA	↓	Destabilizing *WWP1* mRNA	Ma et al., 2018
miR-142	microRNA	↓	Destabilizing *WWP1* mRNA	Tu et al., 2017; Wang et al., 2021
miR-584-5p	microRNA	↓	Destabilizing *WWP1* mRNA	Li et al., 2017
SNHG12	Long non-coding RNA	↑	Sponging miR-129-5p and stabilizing *WWP1* mRNA	Li et al., 2019
circWAC	Circular RNA	↑	Sponging miR-142 and stabilizing *WWP1* mRNA	Wang et al., 2021

## Protein Structure and Activity Regulation of WW Domain-Containing E3 Ubiquitin Protein Ligase 1

**FIGURE 2:**
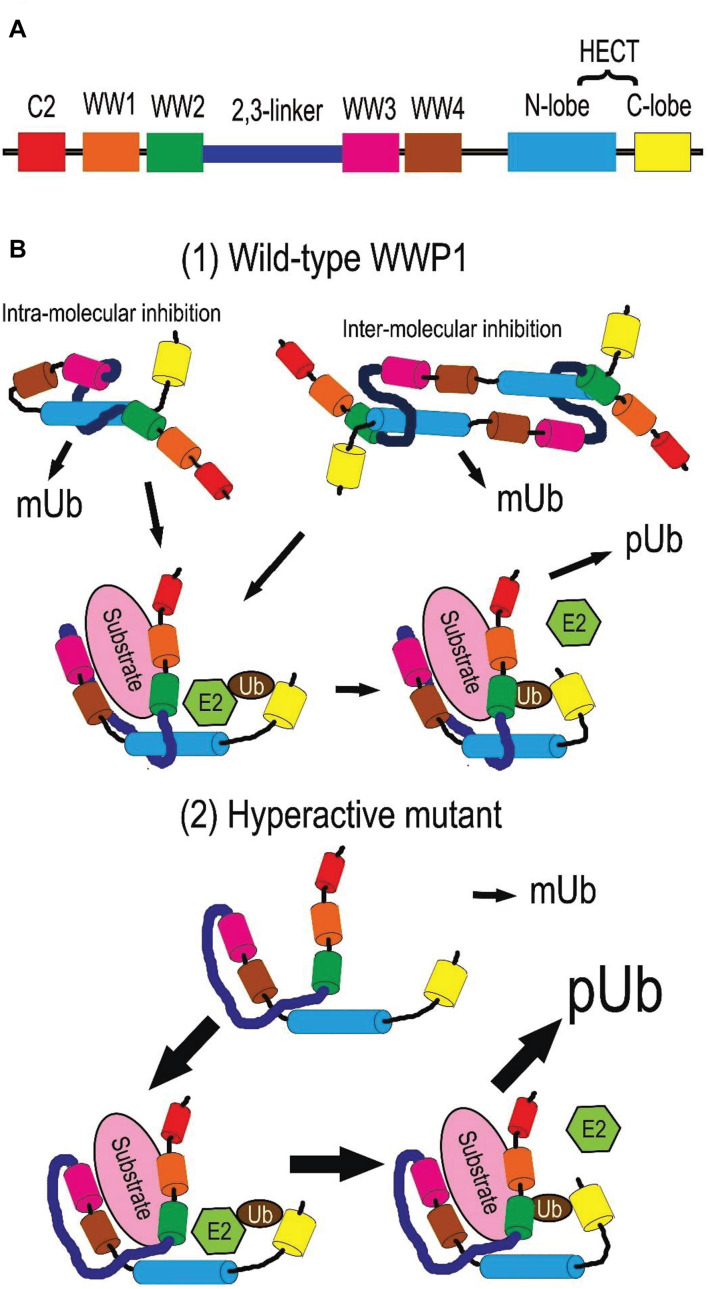
Protein structure and activity regulation of WWP1. **(A)** Depiction of WWP1 protein structure. Each domain is depicted in a certain color (the same below). **(B)** Activity regulation of WWP1. (1) Wild-type WWP1 is autoinhibited via intra- or inter-molecular interactions in a steady state: the HECT domain is sequestered by the 2,3-linker and WW2∼4 domains. In this state, WWP1 has the monoubiquitination (mUb) activity to modify itself and some substrates. Binding of a substrate can partially disrupt the inhibitory interactions and release WW domains from N-lobe. This endows WWP1 with moderate polyubiquitination (pUb) activities: the N- and C-lobes collaborate to transfer ubiquitin chains (Ub) from E2 ligases sequentially. (2) Some cancer-related mutations severely break the autoinhibitory interactions and generate hyperactive WWP1 proteins, which induce elevated polyubiquitination of some substrates.

Due to alternative splicing post transcription, the *WWP1* gene generates at least 6 isotypes of protein in homo sapiens (Flasza et al., 2002; Zhi and Chen, 2012). The predominant isoform is the longest one, which encompasses 922 amino acid residues. Without special instructions, WWP1 refers to this isotype in general. It is comprised with an N-terminal Ca^2+^-dependent lipid-binding (C2) domain, four WW domains (WW1∼4) in the middle, and a homologous to the E6-AP carboxyl terminus (HECT) domain (as depicted in Figure 2A). The structures and functions of other isoforms need further study. The C2 domain is responsible for protein-protein interaction and membrane targeting (Plant et al., 1997; Wiesner et al., 2007; Wang et al., 2010). Each WW domain contains 35∼40 residues in a triple strand β-sheet structure, which is characterized by two tryptophan (W) residues spaced 20∼22 residues apart. It is well known that the WW domains mediate the interaction with diverse substrates or adaptors, especially those containing PY motifs (Sudol et al., 1995; Mosser et al., 1998; Sudol and Hunter, 2000; Li Y. et al., 2008; Li et al., 2009). There is an autoinhibitory link, named 2,3-linker, between the second and the third WW domains (Wang et al., 2019). The HECT domain at the C-terminus endows WWP1 with E3 ligase activity. It can be divided into two lobes: the N-lobe can bind to an E2 enzyme such as UbcH5 and UbcH7, while the C-lobe is involved in the ubiquitination (Ub) process (Verdecia et al., 2003). The cystein 890 residue (C890) in the C-lobe is critical for ubiquitin transferring, because it can form a covalent bond with ubiquitin (Seo et al., 2004). There is a flexible hinge loop between both lobes, which can bend to execute the sequential addition of ubiquitin from E2 to the substrates (Verdecia et al., 2003; Lorenz et al., 2013).

WW domain-containing E3 ubiquitin protein ligase 1 can be post-translationally autoregulated to modulate its stability and E3 ligase activity. Ceshi Chen group reported that WWP1 protein undergoes autoubiquitination and proteasomal degradation (Chen and Matesic, 2007). Céline Prunier group found that an intra-molecular interaction between the C2 and/or WW and HECT domains of WWP1 makes WWP1 self-catalyze its monoubiquitination (mUb) at steady states, leading to stabilization of WWP1 and silence of its activity to polyubiquitinate (pUb) its substrates such as TGFβ type I receptor (TβR1). The intra-interaction is disrupted upon binding to the complex composed of TβR1 and Smad7, the latter of which is a bridging adaptor between TβR1 and WWP1. This switches the mUb activity of WWP1 toward a pUb activity, thereby driving the degradation of WWP1 itself as well as of TβR1. Removal of the WW domains can also convert auto-mUb of WWP1 to auto-pUb with both K48- and K63- linked polyubiquitin chains, which thereby facilitate proteasomal and lysosomal degradation of this E3 ligase (Courivaud et al., 2015). The replacement of a glutamate by a valine at position 798 (E798V) leads to constitutive pUb and degradation of WWP1 via disrupting this intra-interaction (Courivaud et al., 2015). This hyperactive mutant can cause prostate cancer in human (Chen et al., 2007a; Courivaud et al., 2015).

A multi-lock inhibitory mechanism for fine-tuning activity of WWP1 was recently proposed by Wenyu Wen group. Their data demonstrate that C2 domain cannot form a stable complex with HECT, and deletion of C2 domain alone or together with WW1 has little impact on the ligase activity. On the other hand, removing WW2/3/4 leads to a partial activation of WWP1, while deletion of the 2,3-linker strikingly increases WWP1 activity, indicating different importance of the WW and 2,3-linker domains in autoinhibition. Further, their data suggest that WWP1 employs the 2,3-linker, WW2∼4, and HECT domains to form a multilevel inhibitory machinery for tuning its enzymatic activity: WW2 and 2,3-linker interact with HECT; Tyr543 (Y543) in the HECT domain occupies the canonical PY motif-binding site of WW4; WW2 and WW3 stabilize both termini of 2,3-linker (Wang et al., 2019). Thus, WWP1 is locked in an inactive state by preventing ubiquitin transfer from an E2 ligase. When WW2 and WW4 are engaged by substrates or adaptors, especially those containing PY motifs, they can dissociate from HECT. This leads to a partial activation of WWP1 polyubiquitination activity. In some cases, the 2,3-linker is released from HECT for phosphorylation; dissociation of the WW domains and tyrosine/threonine phosphorylation on the 2,3-linker may cooperate to induce a fully active state of WWP1 (Riling et al., 2015; Grimsey et al., 2018; Jiang et al., 2019; Wang et al., 2019). Phosphorylation of Y543 in HECT may also elevate the ligase activity of WWP1. Due to the key roles of the abovementioned domains in autoinhibition, a significant number of cancer-related mutations of WWP1 are located in these domains, which may impair the autoinhibition and generate hyperactive variants of WWP1 (Wang et al., 2019).

Data from Yu-Ru Lee et al. indicate that the interaction between the 2,3-linker region and the HECT domain can be mediated by either homodimerization or intra-interaction, both of which lead to an autoinhibition of WWP1 E3 ligase activity. Two germline point mutations in the N-lobe, K740N and N745S, may hyperactivate the polyubiquitination activity of WWP1 (Lee et al., 2020).

Generally speaking, the manner of interaction between an E3 ligase and a substrate is a major determinant of the ubiquitination type. EGFR pathway substrate clone 15 (EPS15) does not have a PY motif. It employs its ubiquitin binding motif 2 (UIM2) to recruit WWP1, which monoubiquitinates EPS15 (Woelk et al., 2006). UIMs usually bind ubiquitin with low affinities and fast dissociation kinetics, which make the substrate dissociate from the E3 ligase before a second round of ubiquitin addition occurs (Hicke et al., 2005; Hoeller et al., 2006; Ramanathan and Ye, 2012). Another monoubiquitination substrate of WWP1, p53, has no PY motif either (Laine and Ronai, 2007). Although RNF11 has a PY motif, it binds to the WW1 or WW3 domains of WWP1, instead of WW4 (Chen et al., 2008). All these associations may fail to disrupt the intra- or inter-molecular interaction between the WW4 domain and Y543 of the N-lobe. This may account for WWP1-mediated monoubiquitination of these proteins as well as of WWP1 itself. In the future, the exact binding sites of WWP1 with different substrates is worthwhile to map. On the other hand, how WWP1 mediates different types of polyubiquitination, i.e., K27-, K48-, or K63-linked ubiquitination, is poorly investigated.

Based on the reports mentioned above, we summarize the regulation of WWP1 activity as follows and depict it in Figure 2B. In a steady state, polyubiquitination (pUb) activity of WWP1 is autoinhibited through intra- or inter-molecular interactions: its WW2∼4 domains, especially WW4, sequester the N-lobe of the HECT domain, while the 2,3-linker also binds to the N-lobe. Association of a substrate protein or an adaptor with the WW domains disrupts the autoinhibitory interactions and releases the WW domains from HECT, inducing a partial activity of WWP1 to sequentially deliver ubiquitin chains (Ub) to the substrate. Some factors, e.g., some mutations in the autoinhibition-related domains, can severely break these interactions and release both the WW domains and the 2,3-linker, inducing a fully active WWP1, which aberrantly increases polyubiquitination of some substrates. On the other hand, some substrates recruit WWP1 with loose association manners, which may maintain the abovementioned interactions between domains of WWP1. These substrate proteins, as well as WWP1 itself, are consequently monoubiquitinated by WWP1, even in an autoinhibited state.

## WW Domain-Containing E3 Ubiquitin Protein Ligase 1-Mediated Protein Ubiquitinations and Their Roles in Tumorigenesis

WW domain-containing E3 ubiquitin protein ligase 1 can directly add monoubiquitin or different polyubiquitin chains to a variety of substrate proteins and consequently modulate their stability, trafficking, or functions (Seo et al., 2004; Chen et al., 2005, 2008; Flasza et al., 2006; Zhi and Chen, 2012). These WWP1-mediated modifications affect tumorigenesis through kaleidoscopic pathways. In Table 2, we list the main substrates of WWP1 that we already know, and the signaling axes they involved, as well as the effects of these modifications on tumorigenesis. Though the ubiquitination (Ub) types of some substrates are yet to be identified, most degradation-related modifications are probably K48-linked polyubiquitination (K48 pUb), since K48-linked polyubiquitin chains are the canonical labels recognized by 26S proteasome for degradation (Grice and Nathan, 2016).

**TABLE 2:** WWP1-mediated protein ubiquitinations and their effects on tumorigenesis.

Substrate	Ub type	Effects on substrate	Effects of the ubiquitination on tumorigenesis	References
TβR1	pUb	Degradation		Komuro et al., 2004;
Smad2	pUb	Degradation		Seo et al., 2004;
Smad4	pUb	Degradation		Moren et al., 2005
			ImpairedTGFβ signaling and growth arrest of cancer cells, especially PCa cells	
CK2β	pUb	Degradation	Enhanced TGFβ-induced EMT and metastasis of NSCLC cells	Kim et al., 2018
			Increased survival of PCa and BrCa cells via upregulation of HER2 and EGFR	
RNF11	mUb	Activity repression		Chen et al., 2007a
		Decrease in association and co-localization with EGFR-containing endocytic vesicles		
EPS15	mUb		Unknown	Savio et al., 2016; Pascolutti et al., 2019
	K63 pUb	Enhanced recycling and protein stability	Increased stemness of NSCLC via activation of PI3K-AKT and Ras-ERK axes	Sharma et al., 2007; Yu et al., 2020
EGFR				
HER4	pUb	Degradation	Enhanced mammary epithelial cell proliferation and survival	Feng et al., 2009; Li et al., 2009
	K27 pUb	Inhibition of dimerization and membrane localization	Increased morbidity ofoligopolyposis, CRC, and PCa via derepression of PI3K-AKT-mTOR axis and	Lee et al., 2019, 2020
PTEN				
	K27 pUb	Stabilization and translocation to actin-rich projections	Enhanced invasion/metastasis of BrCa cells via upregulation of WNT-PCP pathway	Nielsen et al., 2019; Zhao et al., 2021
DVL2				
	mUb and pUb			
		Stabilization and translocation to cytoplasm		Laine and Ronai, 2007; Levine and Oren, 2009
p53			Inhibition of p53 tumor-suppressive activities	
				Li Y. et al., 2008;
TAp63α	pUb	Degradation	Survival and migration of cancer cells	Li et al., 2013;
	K63 pUb			
ΔNp63α		Degradation	*Inhibition of cell survival*but upregulation of cell migration	Chen et al., 2018
ΔNp73	pUb	Degradation	*Enhanced apoptosis in HeLa human cervix adenocarcinoma cells*	Chaudhary and Maddika, 2014
KLF2	pUb	Degradation	Unknown	
	K48 pUb			
KLF5		Degradation	*Inhibition of cell survival and metastasis in BrCa and PCa*	
				Zhang et al., 2004; Chen et al., 2005; Dong and Chen, 2009
	K48 pUb			Cao et al., 2011; Sanarico et al., 2018
p27		Degradation	Cell proliferation and AML growth	
LATS1	pUb	Degradation	BrCa cell proliferation	Yeung et al., 2013
JunB	pUb	Degradation		
Runx2	pUb	Degradation		
			*Inhibition ofbone metastasis of BrCa and PCa tumors and cells*	
				Shen et al., 2006; Zhao et al., 2011; Jones et al., 2006; Shu et al., 2013

*Ub, ubiquitination; pUb, polyubiquitination; mUb, monoubiquitination; EMT, epithelial-mesenchymal transition; PCa, prostate cancer; BrCa, Breast cancer; NSCLC, non-small cell lung cancer; CRC, colorectal carcinoma; AML, acute myeloid leukemia. The tumor-suppressive effects of WWP1-mediated ubiquitination are in an italic font in the 4th column.*

Like its homologues, Smad ubiquitination regulatory factors (SMURFs), WWP1 mediates polyubiquitination of several Smad proteins. Seo et al. (2004) found that WWP1 specifically interacts with Smad2, 3, 4, 6, and 7, which are key components of TGFβ signaling pathway (Colak and Ten Dijke, 2017). WWP1 associates with Smad7 to induce ubiquitination and subsequent proteasomal degradation of the TGFβ type 1 receptor (TβR1). On the other hand, WWP1 can mediate ubiquitin-dependent degradation of Smad2 in the presence of TG-interacting factor (TGIF), which is a TALE homeodomain protein and a transcriptional repressor (Seo et al., 2004). Furtherly, Run Shen et al. reported that Smad6 is involved in runt-related transcription factor 2 (Runx2) degradation mediated by WWP1 as well as by its homologues, SMURF1 and SMURF2 (Shen et al., 2006; Li X. et al., 2008). In parallel, Schnurr-3 (Shn3) can also recruit WWP1 to mediate Runx2 degradation (Jones et al., 2006). Moren et al. (2005) found that WW and HECT domain-containing ligases, including SMURF1, SMURF2, WWP1, and NEDD4-2, ubiquitinate and degrade Smad4 in the presence of Smad6 or Smad7. In these scenarios, Smad6, Smad7, Shn3, and TGIF function as adaptors for ubiquitination of other proteins (TβR1, Smad2, Smad4, and Runx2) mediated by WWP1 and its homologous E3 ligases. Via ubiquitinating the abovementioned proteins, WWP1 inhibits TGFβ-induced transcriptional responses and growth arrest in either normal kidney cells, including 293 and MDCK cells (Seo et al., 2004), or PC-3 prostate cancer cells (Chen et al., 2007a). In addition, WWP1 may be involved in downstream effects of TGFβ pathway. Kunhong Kim group reported that WWP1-mediated polyubiquitination and degradation of Casein kinase regulatory subunit, CK2β, is required for TGFβ-induced epithelial-mesenchymal transition (EMT) and metastasis of non-small cell lung cancer (NSCLC) cells via enhancing CK2 activity (Kim et al., 2018). Together, these data demonstrate that WWP1 can affect TGFβ pathway in multi-dimensions to promote neoplasia and progression of several cancer types, such as PCa and NSCLC.

WW domain-containing E3 ubiquitin protein ligase 1 can also regulate epidermal growth factor (EGF) signaling and its downstream pathways such as PI3K-AKT and Ras-ERK, which can promote cell proliferation and chemoresistance (Sharma et al., 2007). Overexpression or hyperactivation of epithelial growth factor receptor (EGFR) can drive formation and progression of multiple tumors, including lung cancer and breast cancer (Sharma et al., 2007). Previous studies suggest that WWP1 regulates EGFRs in different ways. According to data from Ceshi Chen and his colleagues, WWP1 monoubiquitinates RING finger protein 11 (RNF11), which is an E3 ligase mediating polyubiquitination and subsequent proteasomal degradation of EGFR. This RNF11-mediated regulation as well applies in HER2 (human epidermal growth factor receptor 2, also known as ErbB2). They further identified WW1/3 of WWP1 and the PY motif of RNF11 as the binding sites during the modification. This WWP1-mediated monoubiquitination (mUb) impairs RNF11-induced degradation of EGFR and HER2 (Chen et al., 2008). On the other hand, several groups reported that WWP1 monoubiquitinates endocytosis protein EPS15, which is involved in EGFR endocytosis and trafficking. This may subsequently modulate EPS15-mediated endocytosis and degradation of EGFR (Savio et al., 2016; Pascolutti et al., 2019). According to data from Zhuowei Hu group, WWP1 can directly ubiquitinate and upregulate EGFR: WWP1 directly binds to EGFR and induces K63-linked polyubiquitination in the EGFR juxtamembrane region, which enhances EGFR recycling and stability. This consequently upregulates EGFR and activates its downstream signaling pathways as well as stemness of non-small cell lung cancer (NSCLC) (Yu et al., 2020). Ceshi Chen group and Shelton Earp group found that WWP1 can directly ubiquitinate human epidermal growth factor receptor 4 (HER4, also known as ErbB4) and cause its degradation (Feng et al., 2009; Li et al., 2009). It has been reported that HER4 can activate the expression of the tumor suppressor BRCA1 (Muraoka-Cook et al., 2006), as well as the differentiation gene β-casein (Muraoka-Cook et al., 2008). There are also studies demonstrating that HER4 decreases mammary epithelial cell proliferation and survival (Muraoka-Cook et al., 2006; Naresh et al., 2006; Pitfield et al., 2006; Feng et al., 2007; Vidal et al., 2007). Therefore, the role as a negative regulator of HER4 may, to some extent, account for the positive regulation of WWP1 on proliferation and survival of mammary epithelial cells, as well as tumorigenesis of breast cancer (Chen et al., 2007b, 2009; Nguyen Huu et al., 2008). In brief, WWP1 regulates EGFR family proteins either directly or indirectly, leading to enhanced proliferation, survival, or stemness of PCa, BrCa, or NSCLC cells.

Phosphatase and tensin homolog (PTEN) is a classical tumor suppressor that antagonizes PI3K-AKT signaling (Rademacher and Eickholt, 2019). In its dimer configuration at the plasma membrane, PTEN is active to dephosphorylate the D3-phosphate of the second messenger phosphatidylinositol 3,4,5-trisphosphate (PIP3). This leads to a repression of the proto-oncogenic PI3K-AKT signaling pathway, and thus controls cell proliferation, growth, and metabolism (Lee et al., 2019). Data from Pier Paolo Pandolfi group demonstrate that WWP1 mediates K27 ubiquitination of PTEN and inhibits PTEN dimerization and membrane localization. This cytosol monomeric PTEN fails to dampen the growth-promoting signaling cascade consisting of PI3K, AKT, and mechanistic target of rapamycin (mTOR) (Lee et al., 2019). These effects increase the morbidity of oligopolyposis as well as colon and prostate cancers (Lee et al., 2019, 2020). All in all, WWP1-mediated K27 pUb of PTEN may promote tumorigenesis of colon and prostate cancers.

WNT signaling pathways, including the canonical WNT pathway and the WNT-planar cell polarity (WNT-PCP) relay, modulate actin cytoskeleton organization to promote cellular motility (VanderVorst et al., 2019). MacGurn group reported that WWP1 mediates ubiquitination of the WNT signal transducer, disheveled protein 2 (DVL2); this promotes redistribution of DVL2 to actin-rich projections (Nielsen et al., 2019). Another work collaborated by Yingxian Li and Shukuan Ling groups demonstrated that WWP1 mediates K27-linked polyubiquitination (K27 pUb) of DVL2 and subsequently stabilizes it (Zhao et al., 2021). WWP1-mediated ubiquitination of DVL2 initiates the WNT-PCP pathway, resulting in cell motility and breast cancer invasion/metastasis (Nielsen et al., 2019). These data reveal that WWP1-mediated K27 pUb of DVL2 promotes invasion and metastasis of breast cancer via activating WNT-PCP pathway.

p53 family transcription factors, including p53, p63, and p73 proteins, play crucial roles in kinds of cancers. Aaron Laine et al. reported that WWP1 directly binds to p53, though p53 does not have a PY motif. This physical interaction leads to modification of p53 with a monoubiquitin or an unidentified polyubiquitin chain, which intriguingly stabilizes p53 instead of targeting it for degradation. WWP1-mediated modifications also result in nuclear export of p53 and a concomitant decrease in its transcriptional activities (Laine and Ronai, 2007). This is likely to account for WWP1’s oncogenic functions in some cancer types, given that p53 is a key tumor suppressor in most malignancies (Levine and Oren, 2009). Chaudhary and Maddika (2014) reported that WWP1 enhances apoptosis via degrading ΔNp73 in HeLa human cervix adenocarcinoma cells. According to data from our group and Ceshi Chen group, α isoforms of p63 (i.e., TAp63α and ΔNp63α) are polyubiquitinated by WWP1 and consequently targeted for degradation, which can be antagonized by isomerase Pin1 (Li Y. et al., 2008; Li et al., 2013). We speculate that WWP1/Pin1-involved protein stability may modulate p63α-mediated metastasis inhibition, especially in head and neck squamous cell carcinoma (HNSC), where ΔNp63α is the predominant p63 isoform, promoting cell proliferation and growth during the early stage and inhibiting metastasis during the late stage (Chen et al., 2018). Recently, Wang et al. (2019) demonstrated that constitutively active WWP1 promotes cell migration by enhancement of ΔNp63α proteolysis. On the other hand, TAp63α, which is also subject to WWP1-mediated degradation, is well known as a tumor suppressor to arrest cell cycle and inhibit cell migration (Su et al., 2010; Chen et al., 2018). These data indicate that WWP1-mediated ubiquitination of p53 family proteins may possess either oncogenic or carcinostatic functions in different scenarios.

WW domain-containing E3 ubiquitin protein ligase 1 can also polyubiquitinate and destabilize Krüppel-like factors 2 and 5 (KLF2 and KLF5) (Zhang et al., 2004; Chen et al., 2005). KLF5 is a key oncoprotein in breast and prostate cancers, where it promotes cell proliferation, survival, and angiogenesis (Dong and Chen, 2009). Downregulation of KLF5 desensitizes prostate cancer cells to chemotherapy (Jia et al., 2019). Deubiquitination of KLF5 boosts breast cancer cell proliferation and metastasis (Qin et al., 2015; Wu et al., 2019). Though it is well known that WWP1 acts as an oncoprotein in breast and prostate cancers, WWP1-mediated ubiquitination and subsequent degradation of KLF5 indicate that WWP1 functions as a tumor suppressor via dampening KLF5’s positive regulation on cell survival and metastasis of these tumors under some circumstances (Chen et al., 2005). YAP and TAZ, both of which are components Hippo pathway, can inhibit WWP1-mediated ubiquitination of KLF5 via competitively binding to the PY motif of KLF5. As a result, KLF5 is stabilized by YAP or TAZ, leading to enhanced proliferation and survival of breast cells or breast cancer cells (Zhao et al., 2012; Zhi et al., 2012). These data indicate that WWP1-mediated KLF5 degradation, which can be antagonized by YAP or TAZ, may inhibit tumorigenesis of breast and prostate cancers.

In addition, WWP1 is involved in tumorigenesis via ubiquitinating other proteins and targeting them for proteasomal degradation. The cyclin/CDK protein kinase inhibitor, p27, can be ubiquitinated by WWP1 (Cao et al., 2011), resulting in cell proliferation and growth of acute myeloid leukemia (AML) (Sanarico et al., 2018). The large tumor suppressor 1 (LATS1) is a key serine/threonine kinase in the Hippo signaling pathway. Xiaolong Yang group found that WWP1 promotes LATS1 degradation through polyubiquitination and the 26S proteasome pathway. This WWP1-mediated degradation of LATS1 increases cell proliferation in breast cancer cells (Yeung et al., 2013). Data from Lianping Xing group indicate that WWP1 may inhibit bone metastasis of prostate and breast cancer cells via destabilizing chemokine receptor CXCR4 as well as transcription factors Runx2 and JunB (Shu et al., 2013). Both transcription factors can be polyubiquitinated by WWP1 (Jones et al., 2006; Shen et al., 2006; Li X. et al., 2008; Zhao et al., 2011), while it is to be validated whether CXCR4 is a direct substrate of WWP1 (Subik et al., 2012).

## Mutations and Dysregulation of WW Domain-Containing E3 Ubiquitin Protein Ligase 1 in Tumorigenesis

As mentioned above, WWP1 is widely accepted as an oncoprotein and is upregulated in multiple cancer types due to gene amplification (Chen et al., 2007a,b; Nguyen Huu et al., 2008). Besides that, WWP1 can be upregulated at the transcriptional and post-transcriptional levels in cancers. Data from Lee et al. (2019) found that amplified and overexpressed MYC may augment transcription of WWP1 in human prostate cancers (PCa). Wang et al. (2021) reported that circular RNA circWAC acts as a miR-142 sponge to relieve the repressive effect of miR-142 on WWP1, resulting in an upregulation of WWP1 in triple-negative breast cancer (TNBC). Increased expression of WWP1 can lead to polyubiquitination and inactivation of PTEN. This can result in derepression of the PI3K-AKT pathway, which may account for neoplasia of PCa and chemotherapeutic resistance of TNBC (Lee et al., 2019; Wang et al., 2021). According to data from Francesca Bernassola group and their colleagues (Sanarico et al., 2018), in combination with our database analysis (Figure 1B), WWP1 expression is significantly elevated in acute myeloid leukemia (AML) patients and cell lines. Knockdown of WWP1 inhibits AML cell growth and delays leukemogenesis via the accumulation of p27, which is known to be polyubiquitinated and destabilized by WWP1 (Cao et al., 2011; Sanarico et al., 2018). This indicates that high level of WWP1 sustains the growth of AML, to some extent, via inducing p27 degradation. Dysregulation of WWP1 expression and its roles in other cancers needs to be further elucidated.

Numerous mutations have been reported in human cancers, which can cause dysregulation of WWP1 activity. Chen et al. (2007a) identified two sequence alterations, Glu798Val (E798V) and Thr241Ser (T241S), in prostate cancer xenografts. It remains unclear whether the change of T241S has any functional consequence. Glu798 resides on the N-lobe and is critical to the enzymatic activity of WWP1 (Verdecia et al., 2003). Data from Celine Prunier group demonstrate that E798V mutation dramatically boosts polyubiquitination activity of WWP1. This culminates in excessive TβR1 degradation and attenuated TGFβ cytostatic signaling, which may account for tumorigenesis of prostate cancers bearing this alteration (Chen et al., 2007a; Courivaud et al., 2015). Recently, Wenyu Wen group surveyed the COSMIC database and found 159 somatic WWP1 mutations in cancers. 85 of them fall into autoinhibition-related domains, i.e., WW2/3/4, 2,3-linker, and HECT. The authors assumed that these mutations promote oncogenesis via enhancing polyubiquitination activity of WWP1. To prove this hypothesis, they validated several mutations, including R427W, S444L, H517Y, P651A, and E697K, in their study. As expected, they found that all of them significantly increase WWP1 activity. Further, they found that WWP1 mutants facilitate cell migration via promoting ΔNp63α turnover (Wang et al., 2019). According to data from Yu-Ru Lee et al., germline mutations K740N and N745S, which are in the N-lobe of WWP1, can disrupt the 2,3-linker/HECT binding and consequently lead to hyperactivation of WWP1. The hyperactive WWP1 mutant proteins elevate polyubiquitination of PTEN, and result in PTEN inactivation, which in turn triggers hyperactivation of PI3K-AKT-mTOR signaling axis. As a consequence, WWP1 gain-of-function results in a genetic predisposition to oligopolyposis and early onset colon cancers in human individuals, as well as larger xenograft tumors of colorectal carcinoma (CRC) in mice (Lee et al., 2020). However, Philip Cole group recently performed an *in vitro* experiment and found that K740N and N745S mutations do not affect E3 ligase activity, and both mutants show similar dependencies to those of wild-type WWP1 in terms of allosteric activation (Jiang et al., 2021). This discrepancy may be due to the difference between modifications in mammalian cells and *in vitro* catalytic reactions with purified WWP1 from *E. Coli.* These main cancer-related WWP1 mutations are listed in Table 3 in this review.

**TABLE 3:** Validated hyperactive WWP1 mutants.

Mutation	Location in WWP1	Cancer type	Effects on tumorigenesis	References
R427W	2,3-linker			
S444L	2,3-linker			
H517Y	WW4	Breast cancer		Wang et al., 2019
P651A	N-lobe			
E697K	N-lobe			
			Elevated pUb and degradation of ΔNp63α, inducing cell migration and invasion	
K740N	N-lobe			
N745S	N-lobe			
		Colon cancer		Lee et al., 2020
			Aberrant pUb and inactivation of PTEN, enhancing cancer susceptibility	
			Excessive TβR1 pUb and degradation, attenuating TGFβ cytostatic signaling	
E798V	N-lobe	Prostate cancer		
				Chen et al., 2007a; Courivaud et al., 2015

On the other hand, WWP1 is downregulated in several cancer types (Figure 1B) and may play as a tumor suppressor. It is poorly known how WWP1 is downregulated and what exact effects it has on tumorigenesis in these cancer cells. A recent investigation demonstrates that both mRNA and protein levels of WWP1 significantly decline in human glioma tissues and cell lines, compared with normal brain tissues and astrocytes, respectively. Upregulation of miR-30a-5p may lead to this downregulation of WWP1 at mRNA level, and consequently promotes glioma cell proliferation, migration, and invasion via an unknown mechanism (Zhao et al., 2019).

## Conclusion Remarks

To sum up, WWP1 is a cardinal E3 ligase, which mediates ubiquitination of a wide range of proteins. For the most part, WWP1-mediated modifications are K48 polyubiquitination, which promotes proteasomal degradation of substrate proteins. WWP1 also adds monoubiquitin or other types of polyubiquitin chains, which may affect trafficking, localization, lysosomal degradation, and enzymatic activity of the modified proteins. Though functioning as a tumor suppressor under some circumstances, WWP1 is generally accepted as an oncoprotein. Via a plethora of substrates, WWP1 regulates kaleidoscopic signaling pathways such as TGFβ, EGF, WNT, PI3K-AKT, and Hippo pathways, consequently promoting or inhibiting neoplasia and progression of diverse cancers (Table 2). The controversy about WWP1’s effects on tumorigenesis may be due to opposite functions of different substrates, even related proteins (e.g., TAp63 and ΔNp63) or the same substrate (e.g., ΔNp63), as well as different ubiquitination types, in different scenarios.

Owing to gene amplification and regulation by transcription factors such as MYC and p53 as well as a batch of non-coding RNAs (Table 1), WWP1 is prone to overexpress in kinds of malignancies (Figure 1B), especially in breast and prostate cancers (Chen et al., 2007a,b; Nguyen Huu et al., 2008; Li et al., 2009, 2017, 2019, 2020; Tu et al., 2017; Ma et al., 2018; Tao et al., 2018; Lee et al., 2019; Zhao et al., 2019; Wang et al., 2021). No less than 6 WWP1 isoforms have been found in human cells (Flasza et al., 2002; Zhi and Chen, 2012). Different WWP1 isoforms may as well account for discrepant roles of this gene in different tissues or cancer types. Besides the elevated expression levels, enzymatic activation of this E3 ligase is also crucial to formation, growth, metastasis, or chemoresistance of some tumors (Chen et al., 2007a; Wang et al., 2019; Lee et al., 2020). Normally, polyubiquitination activity of WWP1 is tightly controlled via a multi-lock mechanism (Figure 2B). In a steady state, the interactions between autoinhibitory domains make WWP1 merely have the monoubiquitination activity. Binding of some substrates can sequentially disrupt these inhibitory interactions and partially activate WWP1. Some cancer-related mutations can result in abnormally hyperactive WWP1 proteins, which aberrantly increase polyubiquitinations of some oncoproteins or tumor suppressors and consequently promote tumorigenesis (Courivaud et al., 2015; Wang et al., 2019; Lee et al., 2020).

The hyperactive state and oncogenic functions of WWP1 in some cancer types make it possible as a therapeutic target. A recent investigation performed by Lee et al. (2019) found that a natural compound from cruciferous vegetables, indole-3-carbinol (I3C), can inhibit the enzymatic activity of WWP1 and the growth of prostate tumor induced by Hi-MYC in mice. This unravels a potential therapeutic strategy for prevention and treatment of some cancers through WWP1 suppression. In the future, more effective small molecules targeting WWP1 should be explored to make this hyperactive E3 ligase ease off in cancers. However, it should be cautious because WWP1 may as well have tumor-suppressive functions in some scenarios. For instance, though WWP1 overexpression in mammary epithelial cell lines MCF10A and 184B5 leads to increased proliferation (Chen et al., 2007b), knockdown of this E3 ligase promotes migration and bone metastasis of MDA-MB-231 breast cancer cells (Subik et al., 2012). On the other hand, in a same pathway WWP1 can be affected in different ways. For example, in the Hippo pathway, WWP1 executes its tumorigenic function by targeting LAST1 for degradation (Yeung et al., 2013), while two other components of this pathway, YAP and TAZ, antagonize WWP1-mediated degradation of KLF5 and consequently promote growth and metastasis of some breast and prostate cancers (Zhao et al., 2012; Zhi et al., 2012). This may increase the complexity of inhibiting WWP1 as a therapeutic strategy. In conclusion, several factors, including multiple WWP1 isoforms, diverse substrates, different ubiquitination types, and opposite functions of these modifications, may contribute to the complexity of WWP1’s actions in cancers, which should be considered thoroughly to reduce side effects when choosing this E3 ligase as a therapeutic target.

## Author Contributions

LK and YuJ wrote the manuscript. CL and YoJ revised and proofread the manuscript. All authors contributed to the article and approved the submitted version.

## Conflict of Interest

The authors declare that the research was conducted in the absence of any commercial or financial relationships that could be construed as a potential conflict of interest.

## Publisher’s Note

All claims expressed in this article are solely those of the authors and do not necessarily represent those of their affiliated organizations, or those of the publisher, the editors and the reviewers. Any product that may be evaluated in this article, or claim that may be made by its manufacturer, is not guaranteed or endorsed by the publisher.

## Footnotes

1
http://gepia.cancer-pku.cn/
